# Genetic polymorphisms and plasma levels of *BCL11A* contribute to the development of laryngeal squamous cell carcinoma

**DOI:** 10.1371/journal.pone.0171116

**Published:** 2017-02-22

**Authors:** Jian Zhou, Yue Yang, Duo Zhang, Liang Zhou, Lei Tao, Li-Ming Lu

**Affiliations:** 1 Department of Otolaryngology, Eye, Ear, Nose and Throat Hospital, Fudan University, Shanghai, China; 2 Shanghai Key Clinical Disciplines of otorhinolaryngology, Shanghai, China; 3 Shanghai Institute of Immunology, Shanghai Jiaotong University School of Medicine, Shanghai, China; Fondazione IRCCS Istituto Nazionale dei Tumori, ITALY

## Abstract

**Objective:**

We investigated the association between B-cell lymphoma/leukaemia 11A (BCL11A) rs11886868 and rs4671393 polymorphism, plasma BCL11A concentration, and the hazard of developing laryngeal squamous cell carcinoma (LSCC).

**Participants and method:**

In this research, 330 LSCC patients, 310 healthy controls, and 155 vocal leukoplakia patients were genotyped for the BCL11A (rs11886868 C/T and rs4671393 A/G) genotypes by pyrosequencing; the BCL11A concentration was measured using ELISA.

**Results:**

LSCC Patients had a notably higher occurrence of CT at rs11886868 (OR = 2.64, P = 0.025) than the control group; they also had higher GG at rs4671393 (OR = 2.53, P = 0.018). Advanced (III and IV) stage LSCC patients had a notably greater frequency of CT at rs11886868 than those with initial (I and II) stage LSCC (OR = 2.71, P = 0.044 vs. OR = 2.58, P = 0.051). Additionally, there was a 1.59 fold increase in susceptibility for initial stage LSCC related to the G allele (AG/GG) at rs4671393 (P = 0.005); while for patients of advanced stage LSCC the OR was 1.73 (P = 0.002). Moreover, the OR of lymph node metastasis patients at rs4671393 G alleles was 2.41 (P < 0.01); it was 1.38 (P = 0.035) in patients without lymph metastasis. Patients with high incidences of the rs4671393 variation genotype had high plasma BCL11A levels.

**Conclusions:**

BCL11A rs11886868 and rs4671393 genotype variations and correspondingly high BCL11A plasma levels are related to LSCC, besides, differences in plasma levels and genotype distribution may be related to lymph node metastasis status and the stage of LSCC.

## 1. Introduction

LSCC has the 11th highest rate of tumour morbidity in males. The most well-established risk factors for LSCC are alcohol abuse and tobacco use [1]. Squamous cell carcinoma makes up more than 95% of laryngeal malignancies [2]. Management includes chemotherapy and radiotherapy, although at present surgery is the only form of management that reliably lengthens survival [3]. The disease has a poor prognosis, with a five-year survival rate of less than 50%. Local and regional recurrences are the major reason for insufficient progress, especially in stage III and IV patients [4]. Thus, further studies are necessary in order to gain a more specific knowledge of LSCC prognosis and pathogenesis.

Vocal leukoplakia, an epithelial hyperplastic keratosis injury of vocal cords, is usually regarded as a precancerous lesion of LSCC.

BCL11A encodes a transcription factor that was originally found as a retroviral insertion site (Evi9) in myeloid leukaemia cancers of the BXH-2 mouse [5, 6]. BCL11A gene participation in solid cancers has been rarely investigated. Khaled et al. described that BCL11A becomes an oncogene of triple-negative breast cancer and that its overexpression promotes tumour formation [7]. Jiang et al. demonstrated that BCL11A protein expression levels were specifically increased in non-small-cell lung cancer tissues [8]. The study presented here aims to determine whether two functional polymorphisms of BCL11A (rs11886868 and rs4671393) are implicated in an increased risk of LSCC in a Chinese Han population. Here, relationships were studied between plasma BCL11A levels and the genotypes, along with environmental risk factors such as cigarette smoking and alcohol consumption.

## 2. Materials and methods

### 2.1 Ethical statement

This work procedure was permitted by the Medical Research Council of the Eye, Ear, Nose and Throat Hospital, Fudan University, China, (NO. KJ2008-01). The purpose of the study was fully explained to every participant, and written informed consent was obtained at the time of enrolment, in accordance with the World Medical Association Declaration of Helsinki.

### 2.2 Patients and controls

Between October 2012 and August 2014, 330 LSCC patients and 155 vocal leukoplakia patients participated in this work at the Eye, Ear, Nose and Throat Hospital of Fudan University. Healthy volunteers (n = 310) of a corresponding age, ethnicity, and gender were registered as the controls. All patients were Han Chinese and recruited from different areas of China. Informed consent was acquired on the basis of the Declaration of Helsinki. The clinicopathologic results of malignancy were gathered. Entirely pathological categories of laryngeal carcinoma were squamous cell tumours. Smoking behaviours were categorised as: non-smokers (fewer than 100 cigarettes in his/her lifetime); and smokers (more than 20 cigarettes per day for one year or more). Alcohol consumption was categorised as: drinkers (more than 200 mL per day); and non-drinkers. The features of the subgroups are shown in Table 1.

Inclusion criteria of patients: aged 18–80 years; Subjects parents are of Chinese descent by self-report; Intact cognition; patients diagnosed as LSCC or vocal leukoplakia. Exclusion criteria: Declined to participate; Undergoing termination of surgery.

**Table 1:** Distribution of cases and controls according to selected socio-demographic characteristics.

Characteristics	Controls	LSCC	Odds Ratio (95% CI) ^1*^	*P*-value^1*^	Vocal leukoplakia	Odds Ratio (95% CI) ^2*^	*P*-value^2*^
**Age (y)**	60.37± 5.9	61.45±7.2			58.67±7.9		
**Gender**							
Female	15	9			5		
Male	295	321			150		
**Alcohol** consumption							
No	220	144	Referent		68	Referent	
Yes	90	186	3.16(2.3–4.4)	0.000	87	3.13(2.1–4.7)	0.000
**Smoking**							
No	233	102	Referent		53	Referent	
Yes	77	228	6.76(4.8–9.6)	0.000	103	5.88(3.9–8.9)	0.000
**Stage of LSCC (%)**							
Advanced							
III + IV		153 (46.4)					
Initial							
I + II		177 (53.6)					
**Typing of LSCC (%)**							
Glottic type		215(65.2)					
Supraglottic type		112(33.9)					
Subglottic type		3(0.9)					
**Lymph node (%)**							
N_0_		210 (63.6)					
N_1_+N_2_		120 (36.4)					

1*: OR, *P*-value calculated between LSCC and controls with SPSS

2*: OR, *P*-value calculated between vocal leukoplakia and controls with SPSS.

### 2.3 Plasma and DNA extraction

Five millilitres of peripheral blood was acquired from each participant in an EDTA tube and centrifuged at 3,200 rpm for 12 minutes. Plasma was parted from the blood and kept at -80°C within thirty minutes. Approximately 100 ng/μL of genomic DNA was fetched from the blood using the QIAamp DNA blood mini kit (QIAGEN Inc., Valencia, CA, USA).

### 2.4 Analysis of plasma BCL11A levels and BCL11A polymorphisms

Plasma BCL11A levels were tested through ELISA: Human Total BCL11A KIT (FY0208A, Hu Feng Biological Technology Co., China). Rs11886868 and rs4671393 of BCL11A genotypes were analysed by PCR amplification using particularly designed couples of oligonucleotide primers and then direct sequencing (ABI Prism 3730xl DNA sequencer, PE Biosystems, Foster City, USA). For genotyping, the PCR settings were: 98°C for 10s; 55°C for 15s; 72°C for 60s, each for 30 cycles. All tests were processed and interpreted blindly, without knowledge of the control or patient status. The sequences of primers applied in this research were as follows:

Sense 5’- CACTGAACCCCCCACCTACCA -3’

Antisense 5’- CTCCACTCCCCGTACCTTCC -3’

Forward and reverse primers were used respectively for direct sequencing.

### 2.5 Statistical analysis

Environmental factors, demographic characteristics, and gene frequencies of BCL11A in the controls and patients were analysed and compared by χ2 tests. Smoking and alcohol consumption were the key risk factors for LSCC. We dichotomised these factors and studied their effects on LSCC. We evaluated the effects of genotypes using logistic regression analyses, plasma BCL11A, smoking activity, and alcohol consumption. We analysed the data and 95% confidence intervals (CIs) and odds ratios (ORs) using the SPSS statistical package.

## 3. Results

### 3.1 Demographic information

The risk factors and characteristics of the vocal leukoplakia patients, the LSCC patients, and the controls are shown in Table 1. No difference was found among the ages of the vocal leukoplakia patients (58.67±7.9 y), the LSCC patients (61.45±7.2 y), and the controls (60.37± 5.9 y). Alcohol consumption is a hazard factor for vocal leukoplakia (OR = 3.13, 95% CI = 2.1–4.7; P < 0.01) and LSCC (OR = 3.16, 95% CI = 2.3–4.4; P < 0.01). Smoking is also a hazard for LSCC (OR = 6.76, 95% CI = 4.8–9.6; P < 0.01) and vocal leukoplakia (OR = 5.88, 95% CI = 3.9–8.9; P < 0.01). The stage of LSCC, the metastasis status of the lymph node, and the typing of tumour are shown in Table 1.

The genotyping of the rs11886868 of the BCL11A display the occurrence of three genotypes: CC, TT, and CT. C is the normal allele and T is the mutant one. While the genotyping of the rs4671393, the outcomes show the occurrence of three genotypes: AA, GG, and AG. A is the normal allele and G is the mutant one.

### 3.2 Genotype frequency distribution in patients and controls

The distribution of rs11886868 and rs4671393 of BCL11A SNP amongst the LSCC patients, vocal leukoplakia patients, and healthy controls is summarized in Table 2. Our outcomes showed that risk for laryngeal carcinoma is related to the BCL11A genotype polymorphism. The BCL11A genotype that included the T allele at rs11886868 and the G allele at rs4671393 were to be more frequent in patients than in the controls (Table 2). We found a 2.64 fold increase in susceptibility to LSCC with the occurrence of CT at rs11886868 (P = 0.025) and an OR of vocal leukoplakia patients of 3.30 (P = 0.011). In comparison to persons with AA genotype, the OR of LSCC patients with GG at rs4671393 was 2.53 (95% CI: 1.15–5.57, P = 0.018; Table 2); while for vocal leukoplakia patients it was 3.02 (95% CI: 1.00–9.11, P = 0.041; Table 2). Therefore, the BCL11A rs11886868 CT and rs4671393 GG variant increased the risk of developing vocal leukoplakia and LSCC.

**Table 2:** Association between BCL11A genotypes and development of LSCC and vocal leukoplakia.

Genotype	Controls	LSCC			Vocal leukoplakia		
	n = 310	n = 330	OR (95% CI)	*P*-value	n = 155	OR (95% CI)	*P*-value
Rs11886868							
CC	302	310	Referent		144	Referent	
CT	7	19	2.64(1.10–6.38)	0.025	11	3.30(1.25–8.68)	0.011
TT	1	1	0.97(0.06–15.65)	1	0	1.00(0.99–1.00)	1
Alleles							
C	611	639	Referent		299	Referent	
T	9	21	2.23(1.01–4.91)	0.041	11	2.50(1.02–6.09)	0.038
Rs4671393							
AA	19	10	Referent		4	Referent	
AG	121	94	1.48(0.66–3.32)	0.345	43	1.69(0.54–5.24)	0.361
GG	170	226	2.53(1.15–5.57)	0.018	108	3.02(1.00–9.11)	0.041
Alleles							
A	159	114	Referent		51	Referent	
G	461	546	1.65(1.26–2.17)	0.000	259	1.75(1.23–2.49)	0.002

### 3.3 Frequency distribution of genotype and haplotype on basis of stage of tumour

In order to compare genotype and haplotype distribution with different stages of tumour, LSCC patients were categorised into three groups: healthy control, initial (I and II), and advanced (III and IV). We observed a 2.58 fold increase in risk for patients of initial LSCC related to CT at rs11886868 (P = 0.051); and a 2.71 fold increase in risk for patients of advanced LSCC (P = 0.044). We also found a 1.59 fold increase in susceptibility for patients of initial LSCC related to the G allele (AG/GG) at rs4671393 (P = 0.005), whereas for patients of advanced LSCC the OR was 1.73 (P = 0.002). Consequently, advanced LSCC had an observably higher OR than did those with initial LSCC (Table 3).

**Table 3:** Prevalence of BCL11A polymorphism in controls and patients with regard to initial (I and II) and advanced (III and IV) cancer stages.

Genotype	Controls	Initial (I+II)			Advanced (III+IV)		
		n	OR (95% CI)	*P*-value	n	OR (95% CI)	*P*-value
**Rs11886868**							
CC	302	167	Referent		143	Referent	
CT	7	10	2.58(0.97–6.91)	0.051	9	2.71(0.99–7.44)	0.044
TT	1	0	1.00(0.99–1.00)	1	1	2.11(0.13–34.01)	0.541
Alleles							
C	611	344	Referent		295	Referent	
T	9	10	1.97(0.79–4.90)	0.136	11	2.53(1.04–6.18)	0.035
**Rs4671393**							
AA	19	7	Referent		3	Referent	
AG	121	49	1.10(0.44–2.78)	0.842	45	2.36(0.67–8.34)	0.173
GG	170	121	1.93(0.79–4.74)	0.144	105	3.91(1.13–13.54)	0.021
Alleles							
A	159	63	Referent		51	Referent	
G	461	291	1.59(1.15–2.21)	0.005	255	1.73(1.22–2.45)	0.002

### 3.4 Frequency distribution of genotype and haplotype on basis of stage of lymph node metastasis

The relationship of BCL11A rs11886868 and rs4671393 gene variants to the metastasis status of lymph nodes was also considered. No noteworthy relationship of metastasis risk to rs11886868 polymorphism was detected. Nevertheless, the OR for lymph node metastasis in LSCC patients at rs4671393 AG/GG was 2.41 (P < 0.01); for patients without lymph metastasis it was 1.38 (P = 0.035; Table 4). This research showed that the rs4671393 AG/GG genotypes increased the risk of lymph node metastasis in LSCC.

**Table 4:** Influence of BCL11A polymorphism on lymph node metastasis.

Genotype	Controls	Lymph node metastasis (-)			Lymph node metastasis (+)		
		n	OR (95% CI)	*P*-value	n	OR (95% CI)	*P*-value
**Rs11886868**							
CC	302	202	Referent		108	Referent	
CT	7	8	1.71(0.61–4.79)	0.303	11	4.94(1.66–11.62)	0.001
TT	1	0	1.00(0.99–1.00)	1	1	2.80(0.17–45.10)	0.460
Alleles							
C	611	412	Referent		227	Referent	
T	9	8	1.32(0.50–3.45)	0.572	13	3.89(1.64–9.22)	0.001
**Rs4671393**							
AA	19	8	Referent		2	Referent	
AG	121	68	1.33(0.56–3.21)	0.518	26	2.04(0.45–9.31)	0.531
GG	170	134	1.87(0.80–4.01)	0.146	92	5.14(1.17–22.56)	0.017
Alleles							
A	159	84	Referent		30	Referent	
G	461	336	1.38(1.02–1.86)	0.035	210	2.41(1.58–3.69)	0.000

### 3.5 BCL11A plasma levels in patients and controls

**Fig 1:**
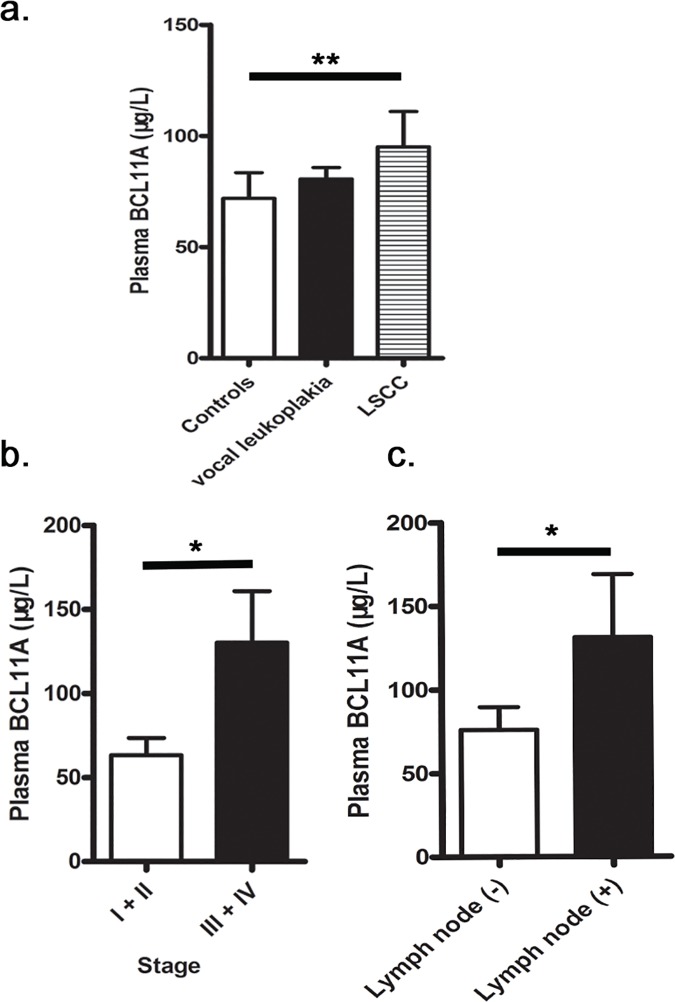
a. Plasma BCL11A concentrations in controls and cases. BCL11A concentrations were analyzed using a standard ELISA and the data were analyzed using the SPSS statistical package. **P<0.01. b. Concentration of plasma BCL11A at different cancer stages. *P<0.05. c. Concentration of plasma BCL11A in patients with and without lymph node.

Differences in BCL11A plasma levels were found in the control group, the LSCC patients, and the vocal leukoplakia patients (Fig 1A). In the LSCC group BCL11A levels (99.36μg/L) were higher than were those in both the healthy controls (71.97μg/L; P < 0.01) and the patients of vocal leukoplakia (80.63μg/L; P > 0.05) (Fig 1A).

### 3.6 Relationship between BCL11A plasma levels and LSCC stage and situation of lymph node metastasis

Different plasma BCL11A levels were detected in patients at different stages of LSCC (Fig 1B); the plasma BCL11A levels were seen to increase with the tumour staging. Plasma BCL11A concentrations at advanced (III and IV) stages (130.24μg/L) were dramatically higher than those at initial (I and II) stages (63.33μg/L, P < 0.05). The plasma BCL11A concentrations in lymph node metastasis LSCC patients (131.22μg/L) were higher than those in no lymph node metastasis LSCC patients (75.99μg/L, P = 0.011; Fig 1C).

### 3.7 Plasma concentrations of BCL11A in relation to BCL11A genotype polymorphisms

**Fig 2:**
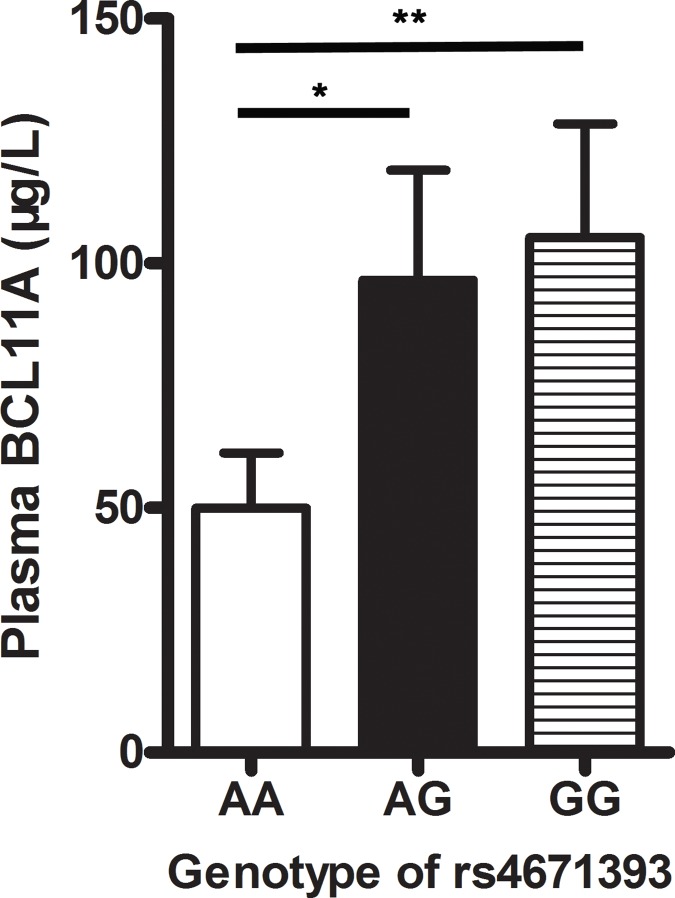
Plasma BCL11A concentrations in different rs4671393 genotypes, *P < 0.05, **P <0.01.

Differences in BCL11A plasma levels were detected in different rs4671393 genotypes (Fig 2). We found that the AG genotype (96.49μg/L) and GG genotype (105.27μg/L) had significantly higher plasma BCL11A concentrations than the AA genotypes (49.90μg/L; P < 0.05 & P < 0.01). Therefore, BCL11A plasma concentrations are consistent with the observation that the BCL11A rs4671393 SNP (AG/GG) genotype are at an increased risk of LSCC.

## 4. Discussion

There is little research on BCL11A genotype polymorphisms, which have been described as related to certain other tumours. As far as we are aware, this study is the first investigation of BCL11A genotype polymorphisms and plasma concentrations in carcinoma.

The BCL11A spans 102 kb on 2p16 and is involved in several tumours [9, 10]. It was first detected in B-cell chronic lymphocytic leukaemia and, moreover, is considered to be a proto-oncogene of malignant haematological diseases [5, 11]. It has been described as displaying limited expression in bone marrow, lymphoid, brain tissue, and foetal liver [12, 13]. Khaled et al. [7] revealed that BCL11A is a novel breast malignance gene and a key regulator in the development of normal mammary epithelium. Our study observed that the plasma BCL11A concentrations (99.36μg/L) of patients of LSCC were dramatically higher than those of both healthy controls (71.97μg/L; P <0.01) and patients of vocal leukoplakia (80.63μg/L; P > 0.05) (Fig 1A); both the BCL11A genotype containing the T allele at rs11886868 and the G allele at rs4671393 were more frequent in the patients than in the healthy controls. Moreover, the BCL11A rs4671393 GG variant increased the risk of developing LSCC and vocal leukoplakia (Table 2).

Some studies observed that the BCL11A-high-expression patients were more frequently diagnosed in the advanced clinical N1/N2 lymphatic metastasis [14–17]. Some studies also showed that BCL11A was a prognostic factor for both overall survival and disease-free survival [8, 18]. In our study, advanced III and IV stage LSCC patients had a dramatically higher BCL11A SNP OR than did those in initial I and II stage LSCC (Table 3); and the rs4671393 AG/GG genotypes increased the risk of lymph node metastasis (Table 4). Moreover, the plasma BCL11A concentrations at advanced stages were significantly higher than those at initial stages; the plasma BCL11A concentrations in lymph node metastasis patients were higher than those in no lymph node metastasis patients (Fig 1B and 1C).

Our study also discovered that smoking and alcohol consumption increased the risk of developing LSCC (OR = 3.16 and 6.76, P < 0.01) and vocal leukoplakia (OR = 3.13 and 5.88, P < 0.01). Consequently, our study showed that environmental factors play an important role in the pathogenesis of vocal leukoplakia and LSCC.

Some evidence suggests that CHEK1 and P21 could be targets of BCL11A. In the beginning of tumorigenesis, BCL11A might act as an oncogene by suppressing P21, CHEK1, and P53, leading to genomic instability and conducing to carcinogenesis[19].

Some limits in this research should also be indicated. Firstly, other pathological types of laryngeal tumour were not taken into consideration. Secondly, the sample size of the study was small. Thus, the results could be chance findings and should be confirmed through larger studies. Therefore, we suggest that further research be conducted to advance and confirm our outcomes.

In summary, the research shows that the rs11886868 C/T and rs4671393 A/G genotypes polymorphisms of BCL11A and increased BCL11A plasma levels are related to risk for vocal leukoplakia and LSCC. Moreover, in patients of advanced laryngeal carcinoma and in those with lymph node metastasis LSCC, a higher OR of rs11886868 C/T and rs4671393 A/G polymorphisms and higher BCL11A plasma levels were discovered. Plasma BCL11A levels were higher in BCL11A rs4671393 SNP (AG/GG) genotype patients.
